# The Relationship between Negative Emotions and Atrial Fibrillation: A Mendelian Randomization Study

**DOI:** 10.31083/j.rcm2510356

**Published:** 2024-10-08

**Authors:** Xiao-Ting Sun, Yu-Qiao Pang, Hui Li, Wu-sha Liuhuo, Chao Tang, Li-Zhi Zhao

**Affiliations:** ^1^Department of Cardiology, The Affiliated Traditional Chinese Medicine Hospital, Southwest Medical University, 646000 Luzhou, Sichuan, China

**Keywords:** atrial fibrillation, negative emotions, Mendelian randomization

## Abstract

**Background:**

The relationship between negative emotions and atrial fibrillation (AF) has garnered significant attention, yet observational studies have yielded contradictory findings regarding the causal associations between the two. Our study sought to provide genetic evidence for a causal relationship between negative emotions and AF through Mendelian randomization (MR) study.

**Methods:**

Utilizing genetic variations associated with negative emotions and AF as instrumental variables (IVs), a two-sample MR study was implemented. The potential causality between the two was initially assessed by using negative emotions as exposure and AF as outcome. Subsequently, potential reverse causality was evaluated by using AF as exposure and negative emotions as outcome. The inverse variance weighted (IVW) method served as the primary analysis for the two-sample MR, supplemented by weighted median method, MR-Egger regression, Simple mode method, and Weighted mode method. Sensitivity analyses were performed using the MR pleiotropy residual sum and outlier test (MR-PRESSO), Cochran Q test, and leave-one-out analysis to ensure the robustness of the results.

**Results:**

The two-sample MR analyses revealed that genetic susceptibility to AF had no potential causal effect on negative emotions (*p* > 0.05). Conversely, genetic susceptibility to negative emotions was positively correlated with an increased relative risk of AF [odds ratio (OR), 1.173, 95% confidence interval (CI): 1.115–1.235, *p* = 8.475 × 10^-10^]. Furthermore, neither horizontal pleiotropy nor heterogeneity was detected in the analysis.

**Conclusions:**

Genetic evidence from the study supports a potential causal link between negative emotions and AF. The study suggests that negative emotions may elevate the risk of AF, and the escalation of negative emotions in AF patients is more likely attributable to modifiable factors rather than genetically related factors.

## 1. Introduction 

Atrial fibrillation (AF), the most prevalent rapid cardiac arrhythmia encountered in clinical settings, can readily result in heart failure, stroke, and even life-threatening conditions. In the last five decades, the incidence of AF has tripled [1]. It is projected that by 2050, at least 72 million people in Asia are expected to be diagnosed with AF as medical technology advances and average life expectancy increases [2]. The symptoms associated with AF, coupled with the increasing prevalence of the AF population, have led to a growing number of individuals seeking healthcare services, thereby creating a substantial socioeconomic burden. Therefore, early detection and prevention strategies are essential for the effective management of AF.

Negative emotions, a pervasive health issue worldwide, encompass depression, anxiety, anger, and sadness, the most common of which are anxiety and depression. Anxiety is the most prevalent negative emotion, with an estimated global prevalence of approximately 7.3% [3]. Depression ranks among the top three leading causes of non-fatal health loss, affecting over 280 million individuals worldwide [4]. Several meta-analyses indicate that anxiety and depression are independent risk factors for cardiovascular disease, with a specific correlation observed between their occurrence and the development as well as adverse outcomes of cardiovascular disease [5, 6, 7]. There exists a complex relationship between negative emotions and AF. Studies suggested that negative emotions may foster an environment conducive to the initiation and perpetuation of AF. Simultaneously, AF can induce varying degrees of negative emotions in patients [8, 9]. Although an increasing number of observational studies are investigating the link between negative emotions and the development of AF, the causal impact of negative emotions on AF remains contradictory [10, 11].

The majority of studies in this field are currently observational studies, primarily due to objective factors such as ethics and research methods. However, these studies can be impacted by confounding factors and reverse causation, which limits their ability to provide strong evidence for a causal relationship between negative emotions and AF. Genome-wide association studies (GWAS) offer a new approach to investigating the genetic basis of diseases by analyzing genetic variants in large populations to determine their association with diseases [12]. Mendelian randomization (MR), a method utilizing genetic variations as instrumental variables (IVs), allows for the assessment of causal links between exposures and outcomes [13]. In MR studies, allelic genes are randomly allocated at conception following Mendel’s second law, and mutations at specific loci occur before the onset of the disease. By effectively reducing biases that may arise in observational studies, MR studies have been widely used to explore potential connections between diseases, to identify new treatment methods and prevention strategies. In light of these considerations, our study uses GWAS data on negative emotions and AF to perform a two-sample MR analysis. This analysis aims to evaluate the association between negative emotions and AF, thereby overcoming the limitations of traditional observational studies and providing clarity on the causal relationship between negative emotions and AF.

## 2. Materials and Methods

### 2.1 MR Design Data Sources

The two-sample MR study must adhere to three core assumptions [14]: ① the relevance assumption: genetic variations must be strongly correlated with exposure; ② the exclusivity assumption: genetic variations have no direct association with outcome and can solely impact the outcome through exposure; and ③ the independence assumption: genetic variations are not related to confounding factors.

**Fig. 1.**
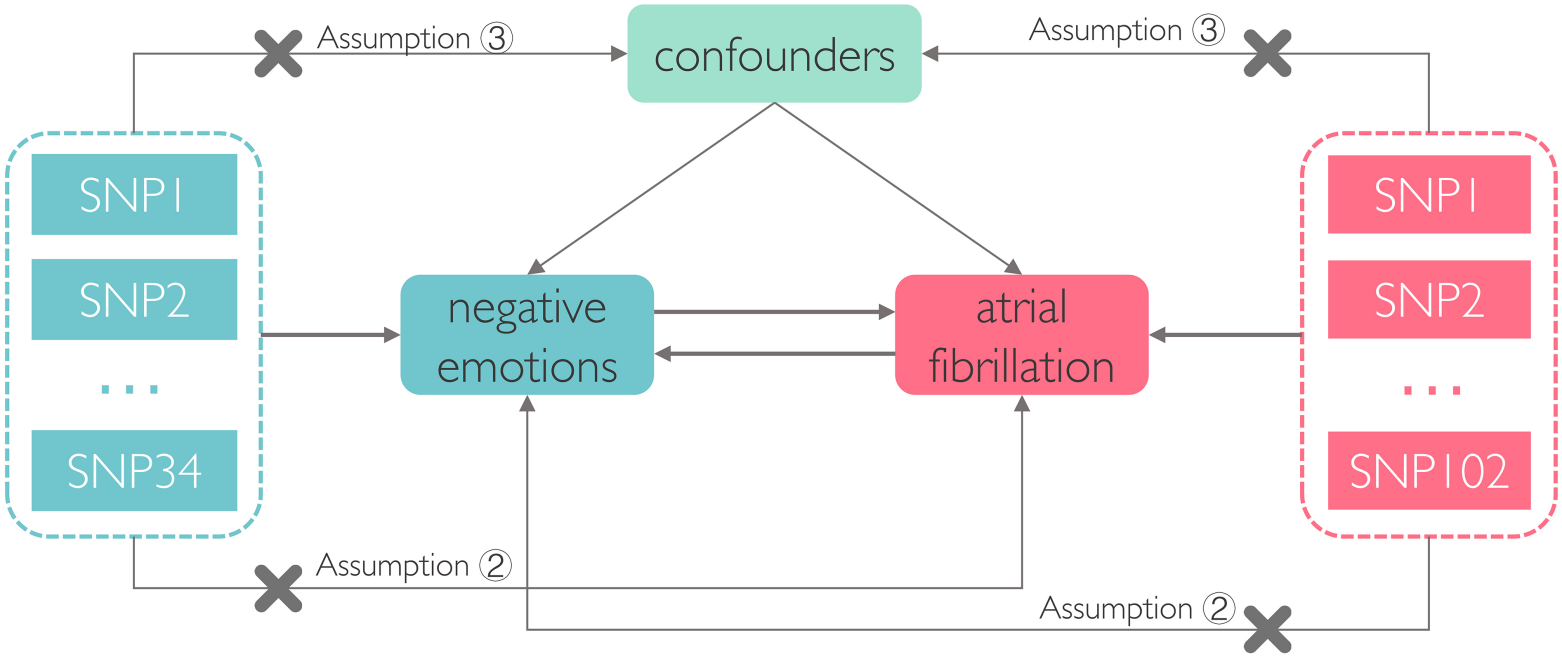
**The flowchart illustrating the analyses for the two-sample Mendelian randomization (MR) study**. Assumption ②, the exclusivity assumption; Assumption ③, the independence assumption; SNP, single nucleotide polymorphism.

Our study utilized published GWAS data on negative emotions and AF and screened single nucleotide polymorphisms (SNPs) that meet the three core assumptions above as IVs for a two-sample MR analysis to uncover the causal link between negative emotions and AF. Considering the potential effect of reverse causation, we initially conducted an MR analysis with AF as the outcome and negative emotions as exposure. Subsequently, by treating AF as the exposure and negative emotions as the consequence, the causal link between AF and negative emotions was evaluated. The study employed the inverse variance weighted (IVW) method as the primary analysis method and simultaneously used Weighted median method, MR-Egger regression, Simple mode method, and Weighted mode method for analysis. Sensitivity analyses were carried out using various methods to ensure the reliability and robustness of the results (Fig. 1).

### 2.2 Data Sources

The exposure and outcome data were extracted from the IEU Open GWAS databases (https://gwas.mrcieu.ac.uk/), and to minimize bias, both samples in this study were sourced from European populations. Specifically, the data for AF genetic variants were obtained from the study conducted by Nielsen *et al*. [15]. After examining genetic variants in 60,620 cases and 970,216 controls from six cohorts, they identified 111 genomic regions significantly associated with AF. This dataset comprises 33,519,037 SNPs and is openly accessible in the IEU Open GWAS database. Similarly, the data for the genetic variants associated with negative emotions were sourced from the IEU Open GWAS database, comprising 459,560 samples and 9,851,867 SNPs. Among these samples, there were 158,565 cases and 300,995 controls. Unfortunately, the negative emotions mentioned here mainly include anxiety and depression, as we did not find useful data regarding other negative emotions such as sadness and anger. All genetic association data for the study are presented in the **Supplementary Table 1**.

### 2.3 Selection of IVs

The best IVs were chosen in accordance with the two-sample MR core assumption to guarantee the authenticity and precision of the causal connection between negative emotions and AF. First, IVs were chosen from SNPs associated with the exposure, and these SNPs needed to meet the following criteria: (i) SNPs were significant loci at the genome-wide level (*p* < 5 × 10^-8^), (ii) the F-statistic was greater than 10 to avoid weak instrumental variable effects (F = ⟦b⁢e⁢t⁢a⟧^2^/⟦s⁢e⟧^2^) [16]. Second, it was essential to ensure that the selected SNPs were independent of each other (parameters: r^2^ < 0.001, kb = 10,000) to mitigate bias from linkage disequilibrium (LD). Third, palindrome SNPs were removed to guarantee that the influence of SNPs both on exposure and outcome originated from the same allele. Fourth, to maintain the core assumptions ② and ③, SNPs directly associated with the outcome were excluded, and those related to confounding factors were discarded using the PhenoScanner database [17]. Finally, the MR pleiotropy residual sum and outlier test (MR-PRESSO) and Egger-intercept method were performed to detect and address potential horizontal pleiotropy, as well as to remove outliers.

### 2.4 Positive Control Analysis

We performed a positive control MR analysis to illustrate the anticipated effect on outcome that has established a causal association with the exposure, thereby supporting the validity of the genetic instruments. Anxiety and depression have been linked to hypertension, so we included hypertension as an additional positive control outcome [18]. We selected ischemic stroke as a positive control outcome for AF, given that AF is the primary underlying cause of this type of stroke [19]. Only exposures showing anticipated associations with these favorable control outcomes were subjected to the primary MR analysis. Genetic association information for these positive control outcomes is available in the **Supplementary Table 1**.

### 2.5 Statistical Analysis

The two-sample MR analysis of the screened SNPs was employed using R software (version 4.2.3; The R Foundation for Statistical Computing, Vienna, Austria) with the two-sample MR package and the MR-PRESSO package [20]. The IVW method was predominantly employed in this study to evaluate the causal correlation between AF and negative emotions. In cases where only one SNP was available, the Wald ratio method was utilized [21]. Additionally, as supplements, Weighted mode method, Simple mode method, MR-Egger regression approach, and Weighted median method were applied. The results were presented as odds ratio (OR), serving as the effect measure to portray the strength of the link between anticipated genetic susceptibility to exposure and the likelihood of outcome.

### 2.6 Sensitivity Analysis

Several sensitivity analyses were conducted to ensure the robustness of the findings. First, the heterogeneity of each SNP estimate was assessed using Cochrane’s Q test. When the Q value was significant (*p* < 0.05), an IVW-based multiplicative random-effects model was employed [22]. Otherwise, a fixed-effects model was utilized. Second, the presence of horizontal pleiotropy among the included SNPs was evaluated using the Egger intercept method and the MR-PRESSO test [23, 24]. If horizontal pleiotropy was detected, outliers were removed before conducting the MR analysis. Finally, in the leave-one-out analysis, each SNP was sequentially removed to examine the MR effects of the remaining SNPs and evaluate the stability of the analysis’s findings.

## 3. Results

### 3.1 SNPs for Negative Emotions

Initially, 44 SNPs associated with negative emotion at a genome-wide significance (*p* < 5 × 10^-8^) were selected. After clumping for linkage disequilibrium (r^2^ < 0.001; distance = 1000 kb), 41 independent SNPs were obtained. Two palindrome SNPs were excluded from the remaining 41 SNPs after combining them with AF GWAS data. Subsequently, five outliers (rs10035449, rs10143492, rs2698323, rs7528182, rs9347903) were excluded through MR-PRESSO analysis. No significant associations with confounding factors were identified among the remaining SNPs based on the PhenoScanner database. Finally, 34 SNPs were selected as IVs, each with an F-statistic greater than 10, indicating the absence of weak instrument bias. Further details are provided in the **Supplementary Table 2**.

### 3.2 Positive Control Analysis

According to the result of the positive control analysis, genetic variations in negative emotions were linked to a higher risk of hypertension (OR 2.327, 95% confidence interval (CI) 1.046–5.176, *p* = 0.038). Additionally, when functional variants of AF were utilized as instruments, the results showed that genetic variations in AF were linked to an increased risk of ischemic stroke (OR 1.003, 95% CI 1.002–1.004, *p* = 2.173 × 10^-15^). To summarize, the positive control analyses supported the validity of the chosen genetic instruments for negative emotions and AF (Further details are available in **Supplementary Table 3**).

### 3.3 Causal Effect from Negative Emotions on AF

**Fig. 2.**
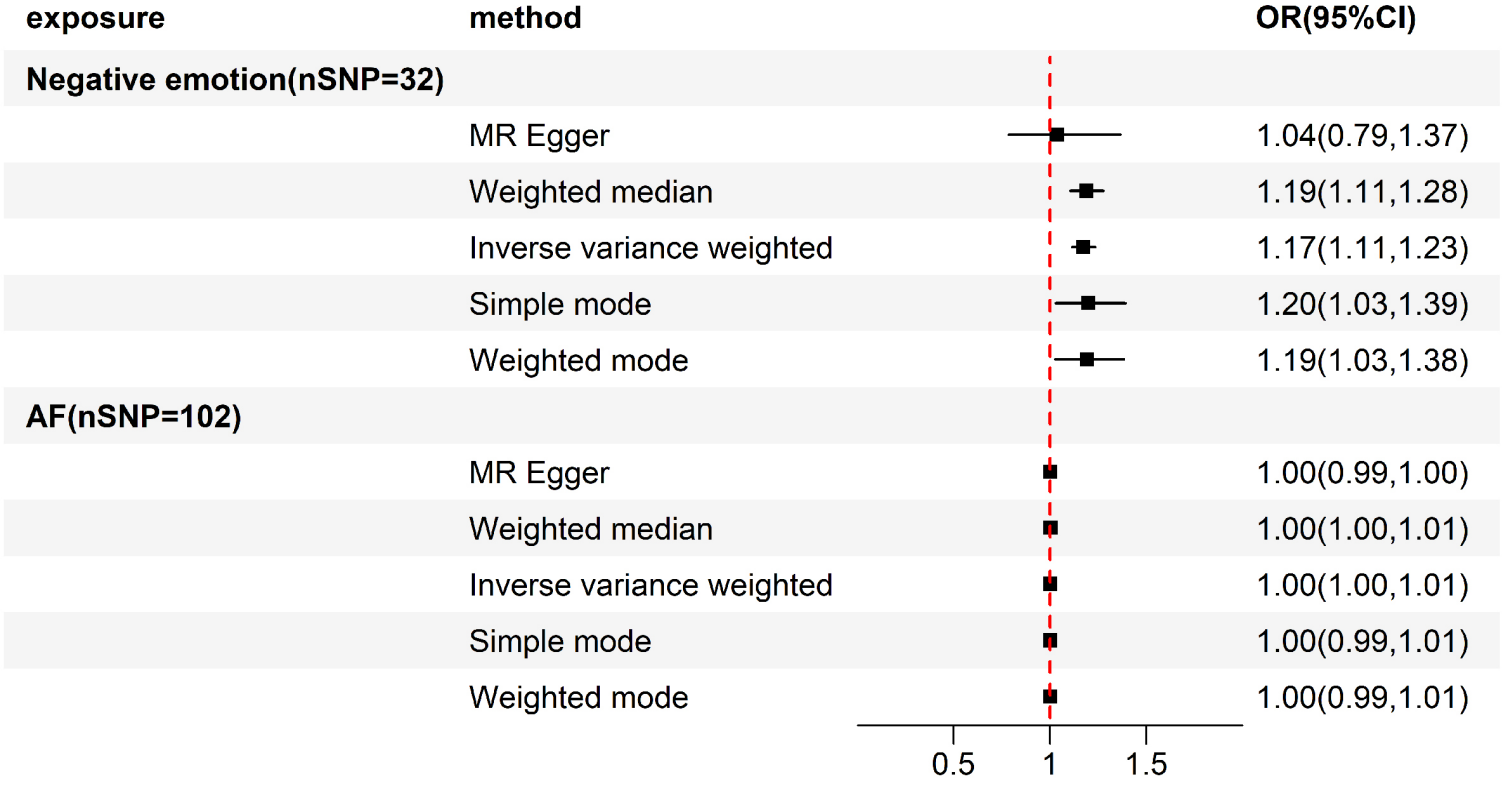
**Forest plot of results from forward and reverse Mendelian randomization (MR) analysis**. Abbreviations: AF, atrial fibrillation; SNP, single-nucleotide polymorphism; OR, odds ratio; CI, confidence interval.

**Fig. 3.**
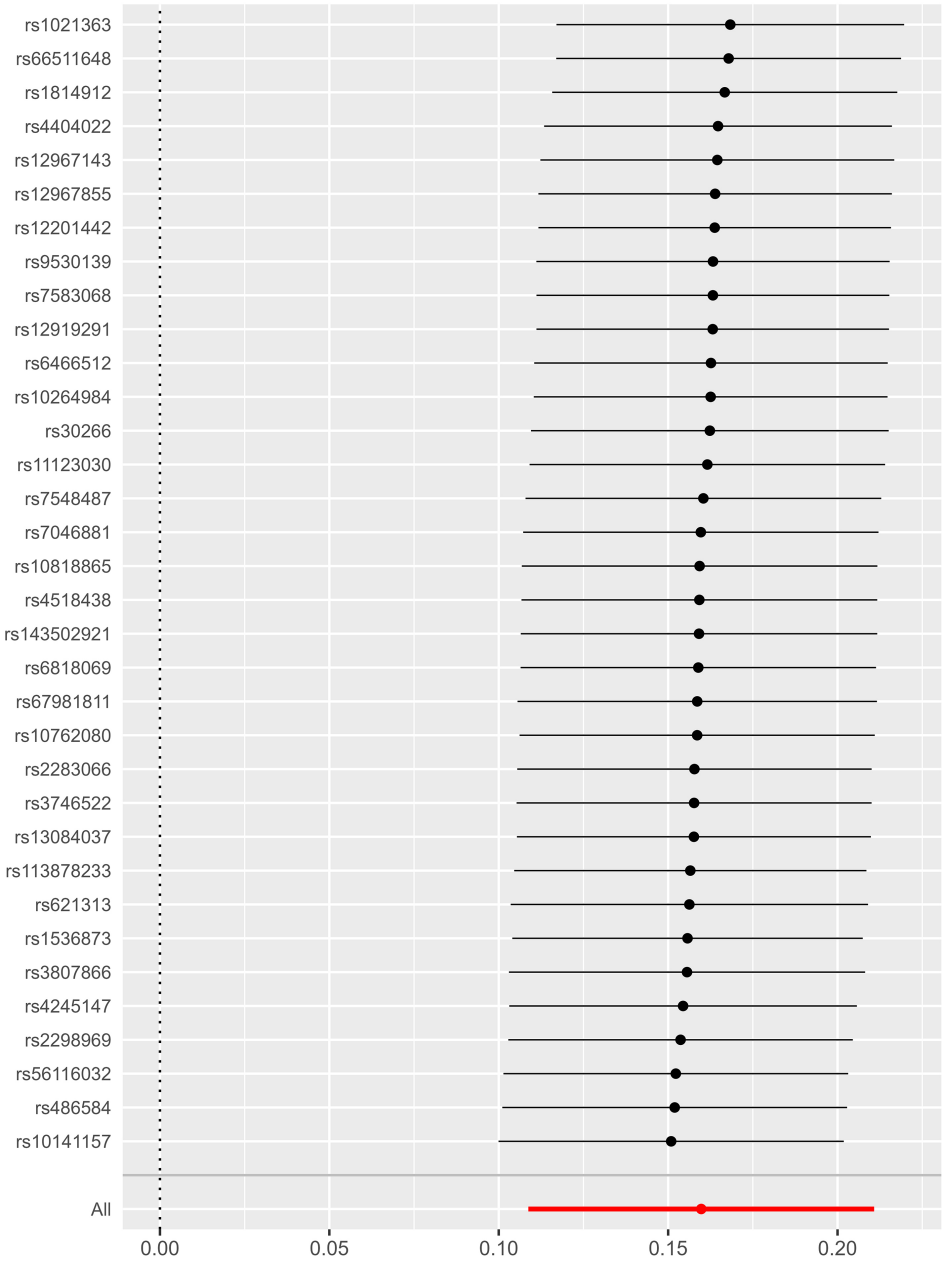
**MR leave-one-out sensitivity analysis for negative emotions on AF**. Notice: The MR estimates for negative emotions on AF using the inverse-variance-weighted (IVW) approach are depicted by circles, while the bars represent the 95% confidence range of the MR values. Abbreviations: AF, atrial fibrillation; MR, Mendelian randomization.

The MR estimates of genetically predicted negative emotion-related features and the risk of AF are presented in Table 1 and Fig. 2. Utilizing the IVW approach, we observed evidence supporting a potential causal association between negative emotions and AF (OR 1.173, 95% CI 1.115–1.235, *p* = 8.475 × 10^-10^ < 0.05). Additionally, the Weighted median method, the Simple mode, and the Weighted mode also indicated a correlation between negative emotions and the occurrence of AF, although the MR-Egger regression did not yield a positive result. According to the MR-Egger intercept (*p* = 0.375) and Cochrane’s Q test (IVW, *p* = 0.417; MR-Egger, *p* = 0.408), sensitivity analysis showed no significant horizontal pleiotropy or heterogeneity. The MR-PRESSO method did not reveal any IVs that were excluded from the final SNPs included in the analysis. Furthermore, we found that no individual instrument could fully account for the impact of negative emotions on AF through leave-one-out analysis (Fig. 3).

**Table 1.** **The result of Mendelian randomization models**.

Outcome	SNP	Methods	OR (95% CI)	*p* value	Heterogeneity	Pleiotropy
AF	34	IVW	1.173 (1.115,1.235)	8.475 × 10^-10^	0.417	
		Weighted median	1.188 (1.104,1.278)	3.841 × 10^-6^		
		MR-Egger	1.036 (0.786,1.365)	8.030 × 10^-1^	0.408	0.375
		Simple mode	1.198 (1.029,1.394)	2.6189 × 10^-2^		
		Weighted mode	1.193 (1.035,1.374)	2.017 × 10^-2^		
Negative emotions	102	IVW	1.002 (0.999,1.006)	0.156	0.074	
		Weighted median	1.002 (0.997,1.008)	0.306		
		MR-Egger	1.000 (0.994,1.006)	0.990	0.073	0.389
		Simple mode	1.002 (0.991,1.013)	0.747		
		Weighted mode	1.001 (0.995,1.007)	0.688		

Abbreviations: AF, atrial fibrillation; SNP, single-nucleotide polymorphism; OR, odds ratio; CI, confidence interval; IVW, inverse variance weighted method; MR, Mendelian randomization.

### 3.4 Causal Effect from AF on Negative Emotions

A reverse two-sample MR analysis was carried out using the same methodology, with AF as exposure and negative emotions as the outcome, to determine whether AF had a causal effect on negative emotions. Using the SNP screening process mentioned above, 102 SNPs highly related to AF were ultimately comprised in the two-sample MR analysis (**Supplementary Table 4**). The results of the IVW method (OR 1.002, 95% CI 0.999–1.006, *p* = 0.156) indicated no proof of a genetic susceptibility to AF being associated with the occurrence of negative emotions. This finding was consistent across other MR approaches, including MR-Egger regression, Weighted median method, Simple mode, and Weighted mode, which were also negative results, indicating that there is probably no causal relationship between genetically forecast characteristics related to AF and the risk of negative emotions. Furthermore, sensitivity analysis did not find significant heterogeneity or horizontal pleiotropy (Table 1, Fig. 2).

## 4. Discussion

Our study assessed the causal link between negative emotions and AF by identifying SNPs from publicly available GWAS data for a two-sample MR analysis. The results indicated that an inherited susceptibility to negative emotions was linked to a heightened probability of developing AF. Interestingly, despite numerous observational studies demonstrating that patients with AF are more likely to experience anxiety and depression [25, 26], our study did not find an inverse relationship. Genetic variants that increased the risk of AF did not correspond to an increased risk of negative emotions. This finding gives us a new perspective, suggesting that modifiable factors rather than hereditary ones may have a greater impact on the increase of negative emotions in patients with AF.

Although there are few researches on whether negative emotions can cause AF, and the evidence is inconsistent, negative emotions like anxiety, depression, and anger have been demonstrated to significantly affect the heart, being considered both independent risk factors and triggers for AF episodes [8, 27, 28]. A meta-analysis showed that anxiety and depression, two frequent negative emotions, were associated with a 10% and 25% increase in the risk of AF, respectively, while anger was linked to a 15% increase in AF incidence [29]. In a 13-year follow-up study involving 6644 participants with baseline data on depressive symptoms and no history of AF, Garg *et al*. [30] found that a Centre for Epidemiological Studies Depression Scale score of ≥16 and using antidepressants was correlated with 34% and 36% elevated risk of AF, respectively. However, no notable association was found between anxiety, tension, or anger and the incidence of AF. Another prospective study, using Doppler tissue imaging (TDI) to measure atrial electromechanical delay (AEMD) in both anxiety patients and healthy participants, discovered that patients with anxiety exhibited a prolonged AEMD, which is a precursor to the occurrence of AF [31]. To explore the involvement of negative emotions in triggering AF among AF patients, Lampert *et al*. [32] tracked the emotional electronic diaries and 24-hour dynamic electrocardiograms of 95 AF patients for one year. The results suggested that negative emotions served as triggers for AF, with the majority of patients being more likely to experience AF recurrence after reporting anxiety, anger, and sadness, whereas positive emotions reduced the likelihood of an AF event by 85%. Most of the literature on the causal association between negative emotions and AF consists of observational studies in nature, with variations in the quantification of negative emotions, sample size, and follow-up time leading to significant differences between these studies. In addition, negative emotions, especially anxiety and depression, manifest overlapping symptoms with cardiovascular disease. Individuals may seek medical attention for symptoms such as palpitations and chest tightness, which can be easily explained by cardiovascular disease before being diagnosed with anxiety or depression. This circumstance poses a challenge in research, as it becomes difficult to accurately determine the chronological order of occurrence between negative emotions and AF, potentially leading to a reverse causal relationship. Compared with conventional research methods, two-sample MR analysis can effectively minimize the effects of confounding elements and reverse causation, providing a more accurate elucidation of the relationship between negative emotions and AF.

Although anxiety, depression, and other negative emotions are prevalent in AF patients, the findings of the current study suggest that genetic variants linked to an increased risk of AF do not correspond to an increased risk of negative emotions. The findings may offer hope, suggesting that the rise in depression among patients with AF could be influenced more by modifiable factors than genetically related ones. Numerous studies have demonstrated that interventions such as patient education, physical exercise, and comprehensive disease management approaches significantly reduce the incidence of anxiety and depression in AF patients [33, 34, 35]. In a randomized controlled trial, Hendriks *et al*. [36] discovered that a novel nurse-led integrated chronic care approach notably improved the quality of life, including reductions in anxiety and depression, among patients with AF compared to conventional care, along with enhancing AF-related knowledge in the nurse-led care group. Similar findings were observed in another study where AF patients were provided with “mobile AF”, a mobile phone app integrating clinical decision support tools, AF-related knowledge, and patient involvement in decision-making to enhance treatment and care for AF patients [37]. AF patients may experience anxiety and depression due to various factors, including fear of surgery, potential medication side effects (such as stomach bleeding or brain hemorrhage), and the condition of AF and its complications. Patients with prolonged negative emotions are prone to autonomic nervous system dysfunction, neuroendocrine disorders, inflammatory responses, and overactivation of the hypothalamic-pituitary-adrenal (HPA) axis [8, 27]. These pathophysiological changes create an environment conducive to triggering and perpetuating AF. Patients with AF complicated by anxiety, depression, and other negative emotions face an increased risk of recurrent AF after radiofrequency ablation, and their quality of life is significantly diminished [38, 39]. Therefore, identifying negative emotions in AF patients and implementing strategies to reduce them may improve clinical outcomes, and patient quality of life, and alleviate the economic burden associated with AF. This necessitates healthcare professionals’ attention to assessing patients’ psychological status, improving the diagnosis and treatment rate of negative emotional disorders such as anxiety or depression, and adopting a more holistic approach to managing AF patients, rather than solely focusing on medications and surgical treatments, as has been the practice in the past.

Despite efforts to minimize the effects of reverse causality and confounding variables, there are additional limitations in our study. Unfortunately, the study cannot elucidate the association between anger/sadness and AF due to the unavailability of GWAS data for these emotions in the database. Furthermore, the applicability of the study’s findings to other demographics remains uncertain, as the GWAS data analyzed were derived from a European population.

## 5. Conclusions

In summary, our study suggests that genetic susceptibility to negative emotions correlates with a heightened risk of developing AF. However, genetic variants linked to increased AF risk do not correspond to an increased risk of negative emotions. The escalation of negative emotions in patients with AF is more likely attributable to the relationship between how patients perceive their disease and how their disease is managed.

## Availability of Data and Materials

The authors confirm that this study analyzed publicly available datasets. These data can be found here: IEU OpenGWAS project (https://gwas.mrcieu.ac.uk/, accessed on 5 August 2023).

## Supplementary Material

Supplementary material associated with this article can be found, in the online version, at https://doi.org/10.31083/j.rcm2510356.
