# Supplementary material for: The Relationship between Negative Emotions and Atrial Fibrillation: A Mendelian Randomization Study
Source: Rev Cardiovasc Med. 2024 Oct 8;25(10):356. doi: 10.31083/j.rcm2510356 (PMC11522760; doi:10.31083/j.rcm2510356)
Supplement: Supplementary file 1 [file 2153-8174-25-10-356-s1.docx]

Supplementary Table 1. Genetic association data for Mendelian Randomization

| trait | Database | GWAS ID | Population | Number of SNPs |
| --- | --- | --- | --- | --- |
| Negative emotions | UK Biobank | ukb-b-6991 | European | 9,851,867 |
| Atrial fibrillation | HUNT, deCODE, MGI, DiscovEHR, UK Biobank, the AFGen Consortium | ebi-a-GCST006414 | European | 33,519,037 |
| Hypertension | FinnGen biobank | finn-b-I9_HYPTENSESS_EXNONE | European | 16,380,466 |
| ischemic stroke | FinnGen biobank | finn-b-I9_STR_EXH_EXNONE | European | 16,380,466 |

Abbreviations: HUNT: The Nord-Trøndelag Health Study; MGI: the Michigan Genomics Initiative.

Supplementary Table 2. 34 SNPs strongly associated with negative emotions

| SNP | Effect  allele | Other  allele | beta | SE | pval | F-statistic |
| --- | --- | --- | --- | --- | --- | --- |
| rs10141157 | C | T | 0.00579658 | 0.00097716 | 3.00E-09 | 35.18944 |
| rs1021363 | G | A | -0.0074074 | 0.00102059 | 3.90E-13 | 52.67739 |
| rs10264984 | T | C | 0.00583058 | 0.000994135 | 4.50E-09 | 34.39797 |
| rs10762080 | G | A | -0.0056262 | 0.000992175 | 1.40E-08 | 32.15528 |
| rs10818865 | G | A | 0.00682631 | 0.00120174 | 1.30E-08 | 32.26643 |
| rs11123030 | C | T | -0.005959 | 0.000977052 | 1.10E-09 | 37.1978 |
| rs113878233 | T | C | 0.0114703 | 0.00204987 | 2.20E-08 | 31.311 |
| rs12201442 | G | A | 0.00985306 | 0.00158252 | 4.80E-10 | 38.76536 |
| rs12919291 | C | G | 0.0072763 | 0.00124931 | 5.70E-09 | 33.92195 |
| rs12967143 | C | G | -0.0076121 | 0.00107088 | 1.20E-12 | 50.52731 |
| rs12967855 | G | A | -0.0068735 | 0.00104168 | 4.20E-11 | 43.54001 |
| rs13084037 | A | G | -0.0065128 | 0.0011683 | 2.50E-08 | 31.0764 |
| rs143502921 | C | G | 0.00885943 | 0.00142321 | 4.80E-10 | 38.75017 |
| rs1536873 | A | G | 0.00555937 | 0.000984187 | 1.60E-08 | 31.90773 |
| rs1814912 | C | T | 0.0062108 | 0.00106149 | 4.90E-09 | 34.23444 |
| rs2283066 | C | T | -0.0057373 | 0.00100863 | 1.30E-08 | 32.35608 |
| rs2298969 | G | A | -0.005359 | 0.000977753 | 4.20E-08 | 30.04098 |
| rs2876520 | G | C | 0.006013 | 0.000987067 | 1.10E-09 | 37.10985 |
| rs30266 | A | G | 0.00765851 | 0.00104038 | 1.80E-13 | 54.18818 |
| rs3746522 | T | C | 0.00730671 | 0.00121043 | 1.60E-09 | 36.43883 |
| rs3807866 | A | G | 0.0074939 | 0.000990903 | 3.90E-14 | 57.1944 |
| rs393488 | A | T | -0.0056414 | 0.000981676 | 9.10E-09 | 33.02447 |
| rs4245147 | T | C | 0.00579555 | 0.000984572 | 3.90E-09 | 34.64929 |
| rs4404022 | T | A | 0.00559119 | 0.000992331 | 1.80E-08 | 31.74647 |
| rs4518438 | C | T | -0.0056611 | 0.000975737 | 6.60E-09 | 33.66194 |
| rs486584 | T | C | 0.00552277 | 0.000977235 | 1.60E-08 | 31.9386 |
| rs56116032 | G | A | -0.006627 | 0.00118115 | 2.00E-08 | 31.47931 |
| rs621313 | G | A | 0.00748553 | 0.000979968 | 2.20E-14 | 58.34737 |
| rs6466512 | A | G | -0.0057834 | 0.000992323 | 5.60E-09 | 33.96772 |
| rs66511648 | C | T | 0.00617337 | 0.00108633 | 1.30E-08 | 32.29394 |
| rs67981811 | G | C | -0.011852 | 0.00153202 | 1.00E-14 | 59.84866 |
| rs6818069 | G | T | 0.00641818 | 0.001125 | 1.20E-08 | 32.54758 |
| rs7046881 | G | T | -0.0054348 | 0.000990133 | 4.00E-08 | 30.12812 |
| rs7548487 | G | A | 0.00880409 | 0.0015217 | 7.20E-09 | 33.47425 |
| rs7583068 | A | T | 0.00626574 | 0.00104958 | 2.40E-09 | 35.63803 |
| rs9530139 | T | C | -0.0076048 | 0.00123719 | 7.90E-10 | 37.78306 |

Abbreviations: SNP, single-nucleotide polymorphism; SE, standard error.

Supplementary Table 3. The results of positive control Mendelian Randomization analysis

| outcome | Methods | OR (95%CI) | P value | heterogeneity | pleiotropy |
| --- | --- | --- | --- | --- | --- |
| hypertension | IVW | 2.327(1.046,5.176) | 0.038 | <0.05 |  |
|  | Weighted median | 2.415(1.032,5.656) | 0.042 |  |  |
|  | MR-Egger | 6.548(0.048,901.368) | 0.460 | <0.05 | 0.679 |
| Ischemic stroke | IVW | 1.003(1.002,1.004) | 2.173×10^(-15) | 0.083 |  |
|  | Weighted median | 1.004(1.003,1.005) | 1.327×10^(-11) |  |  |
|  | MR-Egger | 1.003(1.002,1.005) | 1.831×10^(-5) | 0.076 | 0.603 |

Supplementary Table 4. 102 SNPs strongly associated with atrial fibrillation

| SNP | Effect allele | Other  allele | beta | SE | pval | F-statistic |
| --- | --- | --- | --- | --- | --- | --- |
| rs10141892 | C | T | -0.0452 | 0.0068 | 2.95E-11 | 44.18339 |
| rs10213171 | G | C | 0.091 | 0.0134 | 1.32E-11 | 46.11829 |
| rs10458662 | G | T | 0.0544 | 0.0088 | 6.93E-10 | 38.21488 |
| rs10520002 | A | G | 0.0626 | 0.0113 | 2.85E-08 | 30.68964 |
| rs10520260 | G | A | -0.0457 | 0.0073 | 3.36E-10 | 39.19103 |
| rs10753933 | G | T | -0.0609 | 0.0067 | 9.84E-20 | 82.61996 |
| rs10773657 | A | C | -0.0575 | 0.0103 | 2.54E-08 | 31.16458 |
| rs10804493 | A | G | 0.0558 | 0.007 | 1.63E-15 | 63.54367 |
| rs10821415 | A | C | 0.0821 | 0.0067 | 2.92E-34 | 150.1539 |
| rs10842383 | T | C | -0.0988 | 0.0095 | 2.88E-25 | 108.16 |
| rs11191116 | T | C | -0.041 | 0.007 | 4.42E-09 | 34.30612 |
| rs11264280 | T | C | 0.1347 | 0.0071 | 3.07E-79 | 359.9304 |
| rs11598047 | G | A | 0.1537 | 0.009 | 8.95E-66 | 291.6505 |
| rs11773845 | A | C | 0.1054 | 0.0067 | 2.39E-55 | 247.4752 |
| rs117984853 | T | G | 0.1228 | 0.012 | 1.34E-24 | 104.7211 |
| rs12245149 | A | C | -0.047 | 0.0067 | 1.66E-12 | 49.20918 |
| rs12426679 | T | C | -0.0391 | 0.0067 | 4.95E-09 | 34.05681 |
| rs133885 | A | G | 0.0405 | 0.0068 | 2.22E-09 | 35.47253 |
| rs140185678 | A | G | 0.1659 | 0.0218 | 2.43E-14 | 57.9135 |
| rs1458038 | T | C | 0.0434 | 0.0072 | 1.74E-09 | 36.3341 |
| rs146518726 | A | G | 0.1605 | 0.0207 | 8.27E-15 | 60.11867 |
| rs1563304 | T | C | 0.0644 | 0.0092 | 2.56E-12 | 49 |
| rs17171711 | T | C | 0.1086 | 0.0087 | 1.95E-35 | 155.8193 |
| rs17380837 | T | C | -0.0501 | 0.0072 | 4.80E-12 | 48.4184 |
| rs1838747 | G | A | 0.0391 | 0.0067 | 4.13E-09 | 34.05681 |
| rs1906615 | T | G | 0.3658 | 0.0081 | ###### | 2039.47 |
| rs2031522 | G | A | -0.0436 | 0.0068 | 1.47E-10 | 41.11073 |
| rs2274115 | G | A | 0.0487 | 0.0076 | 1.69E-10 | 41.06111 |
| rs2288327 | G | A | 0.0919 | 0.0089 | 7.26E-25 | 106.623 |
| rs2359171 | A | T | 0.1746 | 0.0086 | 4.65E-91 | 412.1844 |
| rs2540949 | T | A | -0.0659 | 0.0068 | 2.95E-22 | 93.9189 |
| rs2738413 | G | A | -0.0778 | 0.0067 | 2.55E-31 | 134.8372 |
| rs2759301 | A | G | 0.039 | 0.0067 | 5.04E-09 | 33.88282 |
| rs2834618 | G | T | -0.0944 | 0.0112 | 3.41E-17 | 71.04082 |
| rs28387148 | T | C | 0.0741 | 0.0113 | 6.25E-11 | 43.0011 |
| rs284277 | A | C | -0.0422 | 0.0069 | 1.25E-09 | 37.40475 |
| rs2860482 | C | A | -0.054 | 0.0076 | 1.21E-12 | 50.48476 |
| rs28631169 | T | C | 0.0522 | 0.0084 | 5.35E-10 | 38.61735 |
| rs2885697 | T | G | -0.0439 | 0.007 | 2.88E-10 | 39.33082 |
| rs3176326 | A | G | -0.0626 | 0.0085 | 1.42E-13 | 54.23889 |
| rs337705 | G | T | 0.0564 | 0.0068 | 1.63E-16 | 68.79239 |
| rs34080181 | A | G | -0.0446 | 0.0069 | 1.28E-10 | 41.7803 |
| rs34936990 | A | G | 0.1294 | 0.0101 | 2.95E-37 | 164.1443 |
| rs34969716 | A | G | 0.0702 | 0.0078 | 1.60E-19 | 81 |
| rs35544454 | T | A | -0.0589 | 0.0087 | 1.10E-11 | 45.83446 |
| rs35963991 | T | G | 0.0525 | 0.0095 | 2.80E-08 | 30.54017 |
| rs4073778 | A | C | 0.0486 | 0.0067 | 4.96E-13 | 52.61662 |
| rs4587869 | C | G | 0.0716 | 0.0077 | 1.19E-20 | 86.46585 |
| rs4642101 | G | T | 0.0706 | 0.0069 | 2.95E-24 | 104.6915 |
| rs464901 | C | T | -0.0508 | 0.0072 | 1.53E-12 | 49.78086 |
| rs4757877 | G | A | -0.0723 | 0.0078 | 2.93E-20 | 85.91864 |
| rs4935786 | A | T | -0.0463 | 0.0079 | 4.85E-09 | 34.3485 |
| rs4946333 | G | A | 0.0639 | 0.0066 | 5.47E-22 | 93.7376 |
| rs4965430 | G | C | -0.0441 | 0.0069 | 1.26E-10 | 40.84877 |
| rs55734480 | A | G | 0.0548 | 0.0078 | 2.20E-12 | 49.35963 |
| rs55985730 | G | T | 0.0867 | 0.0149 | 5.24E-09 | 33.85834 |
| rs56201652 | A | G | -0.0531 | 0.0075 | 1.74E-12 | 50.1264 |
| rs577676 | T | C | -0.0923 | 0.0067 | 1.62E-43 | 189.7815 |
| rs60902112 | T | C | 0.0445 | 0.0079 | 1.72E-08 | 31.72969 |
| rs62254082 | C | T | 0.0404 | 0.007 | 6.34E-09 | 33.30939 |
| rs62377206 | A | G | 0.0846 | 0.0147 | 8.21E-09 | 33.1212 |
| rs6462079 | A | G | 0.0466 | 0.0076 | 8.79E-10 | 37.59626 |
| rs6546620 | C | T | 0.0602 | 0.0086 | 3.19E-12 | 49 |
| rs6560886 | C | T | 0.051 | 0.009 | 1.49E-08 | 32.11111 |
| rs6580277 | G | A | 0.067 | 0.0079 | 1.64E-17 | 71.92758 |
| rs6596717 | A | C | -0.0404 | 0.0068 | 3.00E-09 | 35.29758 |
| rs6665642 | T | C | -0.062 | 0.0112 | 3.06E-08 | 30.64413 |
| rs6689306 | G | A | -0.046 | 0.0068 | 1.36E-11 | 45.76125 |
| rs6747542 | C | T | -0.0554 | 0.0067 | 1.10E-16 | 68.37068 |
| rs6771054 | C | T | -0.0457 | 0.0068 | 2.42E-11 | 45.16631 |
| rs6790396 | G | C | 0.0627 | 0.0068 | 2.40E-20 | 85.01925 |
| rs67969609 | G | C | 0.0711 | 0.0126 | 1.71E-08 | 31.84184 |
| rs6838973 | T | C | -0.1514 | 0.0067 | 1E-200 | 510.6251 |
| rs6882776 | A | G | -0.0711 | 0.0074 | 9.64E-22 | 92.31574 |
| rs6994744 | C | A | 0.0405 | 0.0066 | 1.09E-09 | 37.65496 |
| rs71454237 | A | G | -0.062 | 0.0084 | 1.78E-13 | 54.47846 |
| rs7172038 | G | T | 0.112 | 0.0089 | 4.78E-36 | 158.3638 |
| rs7224711 | T | C | -0.0365 | 0.0066 | 3.72E-08 | 30.58425 |
| rs7225165 | A | G | -0.0655 | 0.0111 | 3.20E-09 | 34.82063 |
| rs72694603 | T | C | -0.0553 | 0.0072 | 2.26E-14 | 58.99093 |
| rs72700114 | C | G | 0.2021 | 0.013 | 3.29E-54 | 241.6829 |
| rs72811294 | C | G | -0.072 | 0.0106 | 9.67E-12 | 46.13742 |
| rs72966339 | T | C | -0.0616 | 0.0069 | 7.42E-19 | 79.7009 |
| rs73041705 | C | T | -0.0443 | 0.0073 | 1.55E-09 | 36.82661 |
| rs73366713 | A | G | -0.1035 | 0.0099 | 1.53E-25 | 109.2975 |
| rs74500426 | T | G | -0.0921 | 0.0127 | 4.29E-13 | 52.59105 |
| rs74832855 | G | A | 0.1216 | 0.018 | 1.43E-11 | 45.63753 |
| rs74884082 | T | C | -0.0493 | 0.0078 | 3.48E-10 | 39.94888 |
| rs74910854 | G | A | 0.09 | 0.0164 | 4.31E-08 | 30.116 |
| rs7508 | A | G | 0.0711 | 0.0075 | 1.69E-21 | 89.8704 |
| rs7529220 | C | T | 0.0621 | 0.0098 | 1.98E-10 | 40.15421 |
| rs7574892 | A | G | 0.0552 | 0.0067 | 1.98E-16 | 67.87792 |
| rs76097649 | A | G | 0.1151 | 0.0124 | 1.26E-20 | 86.16031 |
| rs7612445 | T | G | 0.0493 | 0.0084 | 4.81E-09 | 34.44572 |
| rs77316573 | T | C | 0.0529 | 0.0089 | 3.26E-09 | 35.329 |
| rs775498 | G | A | 0.0423 | 0.0074 | 1.05E-08 | 32.67513 |
| rs7789146 | A | G | -0.0584 | 0.0087 | 2.12E-11 | 45.05959 |
| rs7915134 | T | C | -0.1168 | 0.0095 | 1.42E-34 | 151.1606 |
| rs79187193 | A | G | -0.1162 | 0.0153 | 3.15E-14 | 57.68055 |
| rs883079 | T | C | 0.0981 | 0.0074 | 2.84E-40 | 175.7416 |
| rs9506925 | T | C | 0.0449 | 0.0075 | 2.72E-09 | 35.84018 |
| rs9953366 | C | T | 0.049 | 0.0073 | 1.82E-11 | 45.05536 |

Abbreviations: SNP, single-nucleotide polymorphism; SE, standard error.
